# Experimental Simulation-Based Performance Evaluation of an SMS-Based Emergency Geolocation Notification System

**DOI:** 10.1155/2017/7695045

**Published:** 2017-08-30

**Authors:** Isibor Osebor, Sanjay Misra, Nicholas Omoregbe, Adewole Adewumi, Luis Fernandez-Sanz

**Affiliations:** ^1^Covenant University, Ota, Nigeria; ^2^Atilim University, Ankara, Turkey; ^3^University of Alcala, Alcalá de Henares, Spain

## Abstract

In an emergency, a prompt response can save the lives of victims. This statement generates an imperative issue in emergency medical services (EMS). Designing a system that brings simplicity in locating emergency scenes is a step towards improving response time. This paper therefore implemented and evaluated the performance of an SMS-based emergency geolocation notification system with emphasis on its SMS delivery time and the system's geolocation and dispatch time. Using the RAS metrics recommended by IEEE for evaluation, the designed system was found to be efficient and effective as its reliability stood within 62.7% to 70.0% while its availability stood at 99% with a downtime of 3.65 days/year.

## 1. Introduction

Mobile health (M-Health) applications as a subset of electronic health (eHealth) is expected to make use of technologies that address healthcare challenges such as response time, access, quality, affordability, matching of resources, and behavioral norms through the exchange of information at reasonable time [1]. Mobile technologies cannot physically carry drugs, doctors, and equipment between locations, but they can carry and process information: coded data, text, images, audio, and video [2]. The various life-threatening issues arising from delayed response to emergency scenes cannot be overlooked. Delays that occur while trying to locate scenes during emergency and accident cases can lead to loss of lives. During emergency calls, the call taker tries to get the location of an emergency scene from the caller and this can cause delay or even provide inaccurate geographical information. Inaccurate geographical information is given particularly if the victim is at an unfamiliar location and/or when the immediate person to save the day has speech or hearing challenge.

Response time is a common measure in benchmarking the efficacy of emergency services. It is seen as the amount of time that it takes for emergency responders like the ambulance service units or fire fighters to arrive at the scene of an incident after the emergency response system has been activated [3]. Due to the nature of emergencies, fast response time is often a crucial component of the emergency service system. The initiation of new response time, performance standards, and call prioritization has also resulted in significant changes for ambulance services and the way the system is being operated. There are general response time standards in many jurisdictions throughout Europe and the Americas [4, 5]. In achieving this, the notification system and its other components must be highly effective and efficient in terms of timeliness [6].

Emergency medical service (EMS) response providers who are also first responders in emergencies are usually operated by hospitals, private ambulance companies, fire service departments, and the police. Automobiles are also being equipped with telematics systems that automatically open up a voice call and provide valuable crash data when a car is involved in an accident [7, 8]. All of these are in an effort to initiate an efficient notification and alert system in emergencies. This is why this study is based on implementing and evaluating the performance of an SMS-based geolocation notification system aimed at improving the response time and efficiency of ambulatory services in cases such as locating accident and emergency scenes.

The objectives of this study are to model an SMS-based emergency geolocation notification system by generating an architecture diagram. The designed system will be implemented and its performance will be evaluated using the reliability, accessibility, and serviceability (RAS) parameters recommended by the Institute of Electrical and Electronic Engineers (IEEE).

The rest of this paper is structured as follows: Section 2 is the review of related works in the literature. In Section 3, the system's design is discussed and the model diagram of the proposed system is presented. In Section 4, the system implementation is discussed as well as the evaluation of the system's performance through case selection and comparative analysis. We also discuss in this section how data was collected and analyzed as well as the interpretation of the results.  Section 5 concludes the paper and gives suggestions for future work.

## 2. Literature Survey

SMS is widely used and universally accessible since even the simplest phone supports it [9, 10]. It has found its way into medicine for delivering mobile-based health interventions especially during emergencies. A number of works were seen in literature addressing the design of SMS notification systems for use during emergencies. In a study by Kumar and Rahman [11], the SMS functionality of a mobile phone was used in the design of a wireless health recording and alert system for use by the elderly or athletes. It was designed to contain a sensor, base, and server unit. In the proposed system, the user is the sender of the precoded emergency SMS, but in this reviewed work, the system is the sender of the SMS as an alert signal to caregivers based on decisions made from data received from a sensor. In a nutshell, it does not possess the capabilities that will make it useful as an emergency notification system except a specialized sensor capable of sensing accidents is utilized. In another study by Kim et al. [12], SMS was used in the development of a personalized text message service program based on lifestyle questionnaires and data from an electronic health record. It was designed specifically to create an SMS service for weight loss in obese patients. This service becomes unsuitable during emergencies for notifications even though SMS was utilized. The Mobile for Reproductive Health (m4RH) program in Kenya also utilizes SMS [13]. The application provides contraception information to young people in Kenya. To engage the system, a user is expected to send precommunicated codes by SMS to the m4RH system. The system automatically responds with related information in a concise format of messages per respective codes. The system also provides a database of available clinics searchable by users. However, the use of this system is unsuited for emergency situations since it is not equipped with the capacity to determine the geolocation of the sender of the SMS containing precommunicated codes.

A study of the work done in [14], termed emergency SMS, focuses on establishing communication with an emergency center and providing brief and useful information about patient via SMS as against the ability of the proposed model to transmit the geolocation of emergency scenes to an ambulance point. In execution, the patient must first configure the application with his or her personal information, brief medical history, the emergency phone number, and mobile phone number of his family doctor and one or more of his relatives (initiated SMS is sent to these people in cases of emergency). This information is then saved in a text file on the mobile phone. The program, when activated or triggered during an emergency (by pressing a precoded key on the mobile phone for a few seconds) automatically sends the preconfigured text file to all the mobile numbers saved in the text file. The present location of the mobile phone sending the message is determined through global positioning system (GPS); this functionality makes it suitable for use during emergencies.

In a study by Vetulani et al. [15], SMS was used to design a crisis situation management system named POLINT-112-SMS. The system is to support information management and to assist a human in decision making during emergency situations. Its architecture consists of an SMS gateway, a natural language processing module, a situation analysis module (SAM) and a dialogue maintenance module (DMM). In crisis situations, the user initiates the system and sends the crisis report by SMS; this is received as text inputs by the system. It then gathers, processes, and interprets the received information. The interpretation of this information notifies the authorized personnel to get to work. This system was designed specifically for use in noisy or unsecured environment where the use of SMS is seen as most appropriate.

In a study by Omoregbe and Azeta [16], a mobile-based medical alert system is presented for managing diseases where adherence to medication is crucial for effective treatment. The system automatically alerts the patient and medical practitioners about information and emergencies via SMS. It also allows users to receive scheduled appointments and medication updates that will facilitate the treatment process. The system however does not provide any geolocation functionality and is unsuitable for reporting emergencies as it is mainly for health information dissemination from health personnel to patients.

**Figure 1:**
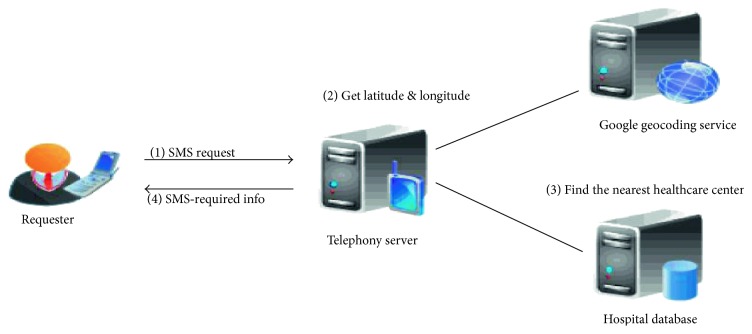
System diagram for locating the nearest healthcare center [17].

Hameed et al. in their study [17] proposed the Medical Emergency and Healthcare Model (MEHM DESIGN). It is enhanced with SMS and MMS facilities focused on incorporating real-time, mobile technology medical emergency systems with a location-based access to the emergency scene. It has a design capacity to utilize both SMS and MMS functionalities. Our concern here is the SMS module of the model, which comes to play when a user in need of medical attention is unaware of the nearest health center. The SMS module was designed to provide three key features and is available only to SMS capable devices. The SMS module was also designed to be responsible for receiving SMS requests from the nearest healthcare center, locating the nearest healthcare center based on input provided by the user, and sending SMS providing information of the nearest healthcare center to the requester as shown in Figure 1().

The E-911 (both phases I and II) [18] was an improvement of the basic 911; this system tries to automatically associate a location with the origin of the call. The caller's telephone number is used in various ways to derive a location that can be used to dispatch police, fire, emergency medical response resources, and other response resources. Automatic location of the emergency makes it quicker to locate the required resources during fires, break-ins, kidnappings, and other events where communicating one's location is difficult or impossible. In a bid not to slowdown response times, wireless E-911 and VOIP-911 were deployed and have helped to overcome the challenge of locating callers by transmitting longitude and latitude information based on the location of the caller's mobile device to the 911 center. The location of cellular callers is determined either by the GPS device within the phone itself or through a network solution that employs triangulation.

The existing systems reviewed in this section all fall short of the feature that makes it possible to automatically transmit geolocations via SMS when using a non-location-based service (LBS) mobile device. This distinguishes it from our proposed system. In summary, the major reason for designing this system is to reduce the time taken by ambulance teams to arrive at emergency scenes using a combination of SMS and geolocation systems in the system design. This approach will help to improve response time to scenes of accidents to save lives and also to bring quick medication/medical help to patients suffering life-threatening ailments especially in developing countries of the Africa continent where mobile phones are very common, but use of internet and GPS is limited. This feature (use of SMS with geolocation triangulation on the GSM network through mobile phones for emergency services) of our system makes it unique in comparison to that of the existing systems.

Records obtained from Federal Road Safety Corps (FRSC), Nigeria, in 2009, state that about 4120 deaths were recorded, with 20,975 seriously injured persons in road transport accidents involving about 11,031 vehicles across Nigeria. In 2008, the commission stated that about 11,341 road transport accidents occurred, claiming a total number of 6661 lives and with 27,980 injured persons. From January to June 2010, road transport accidents amounted to 5560 cases, with 3183 deaths and 14,349 injuries [19]. The uniqueness of the design lies in the aforementioned facts; other similar systems failed to consider the peculiar challenges witnessed in third world countries like those in Africa when it comes to reporting accidents or calling for help. These terrains have sparsely-spread base stations for communication and so limit the use of any form of communication except by SMS. Also, ICT infrastructures are underdeveloped which limits the use of GPS systems, and the safety of patient data being transmitted within heath applications is not guaranteed.

### 2.1. The Worth of Ambulance Response Time in Emergency Medical Services

In EMS, timeliness of care is an imperative subject. The amount of time it takes during an emergency to initiate the appropriate level of care can have a rational effect on patient outcome. This time is known as response time. Response time is a common measurement in standardizing the efficiency and effectiveness of emergency medical services. Paramedic response time to the scene of a call for emergency medical assistance has become a benchmark measure of the quality of the service provided by emergency medical services [20]. In various parts of the world, ambulance services measure their performance using indicators such as response time, on-scene time, and clients' satisfaction [20]. Response time performance and the achievement of response time standards has been the single measure against which the quality of ambulance services has been judged. Response time performance has been used as an indicator of ambulance service quality for many years [21].

Ambulance response time includes the call processing time, team preparation time, and the time it takes to travel to the scene [22]. This can be summarized as the time taken from receiving an emergency call until the time of arrival at the emergency scene. It has also been discovered that response time significantly affects mortality, but not hospital utilization [23].

There are general response time standards in many jurisdictions around the world [21]. These response time standards vary from 7 to 11 minutes in urban areas and 15 to 45 minutes in rural and remote areas [4]. In achieving this response time standards, it was concluded that the notification system with its communication components must be highly effective and efficient in terms of timeliness [6].

There are three different measures of the timeliness of ambulance response and these are the following:

- The amount of time it takes to get to the scene of an emergency, also referred to as the time to scene.
- The amount of time spent at the scene, also referred to as the time at scene.
- The amount of time elapsed from when the ambulance leaves the scene to the time when the ambulance arrives at the hospital, also referred to as the time to hospital.

The scope of this research report covers ways to reduce the amount of time it takes to get to the scene, also referred to as the time to scene. This time to scene can be reduced or lowered through the gathering and storage of addresses and location information more rapidly and precisely for real-time access. Also, if ambulance or ambulatory resources are allocated efficiently, it can also reduce the time to scene.

Some of the major organizational changes and development made in study to the EMS frameworks to facilitate an improved response time performance are [24] as follows:

- Changes to ambulance station designs which include relocations, increase in numbers, and schemes to share premises with other first responders like the fire stations.
- A range of first responder schemes with the emphasis on single-manned vehicles including motorcycles.
- Developments to decrease total call time (receipt of call to vehicle clear for another call) including increased use of standby points and dynamic deployment, improved stocking procedures for vehicles which decrease the need for vehicles to return to stations, and better arrangements with hospitals to enable patients to be taken to the most appropriate hospital rather than the nearest.
- Increased utilization of IT developments including automatic vehicle location, predictive analysis, and data transmission to vehicles (prealert systems).
- Improvements in demand management including demand analysis, predictive analysis, flexible crew rostering, and the use of appropriately trained staffs as first responders.

All these developments come under new capital investment as well as reorganization of existing resources.

## 3. System Design

**Figure 2:**
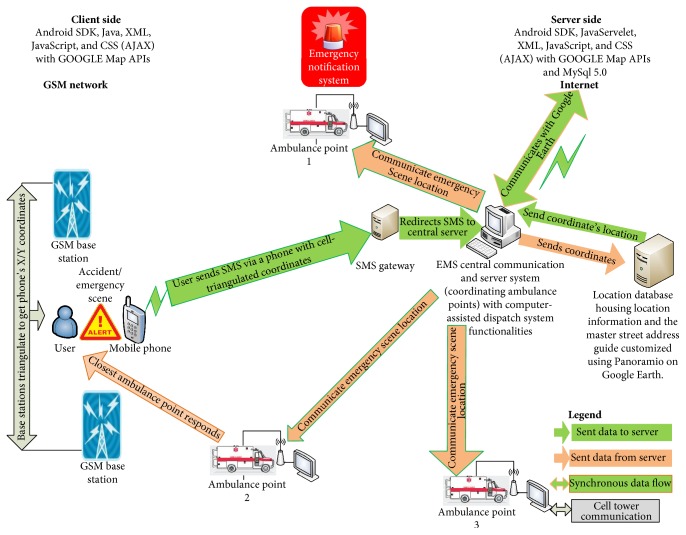
System architecture.

Experimental simulation using artificially created emergency scenarios with the use of mobile phones of different platforms including Blackberry OS, Apple iOS, Android, Symbian, and Windows Mobile was utilized for software and system validation during deployment. As depicted in Figure 2, an EMS-based ambulance service system was set up composing of an EMS point and one central coordinating center with computer-aided dispatching capabilities coded using Android's XML tool and JavaScript (AJAX). Evaluation on the system was carried out using the reliability, availability, and serviceability benchmark. Reliability here is the measure of the system's ability to function correctly, including avoiding data corruption. Availability measures how often the system is available for use, even when its other communication interfaces fail. For example, the server may run forever and so has ideal availability especially during Internet communication connection failure. The key areas analyzed were SMS delivery time analysis and system geolocation and dispatch time analysis.

After setting up the notification system both at the client and at the server side as depicted in Figure 2, an SMS is sent from a location presumed to be an emergency scene. This sent SMS is delivered with all the needed parameters such as latitude, longitude, cell ID, and timestamp which will allow a mapping software on the central server that has been setup in another location to determine the origin of the SMS with the aim of locating and dispatching the closest rescue team to the origin of the SMS. The operating mechanics of the system is outlined as follows:

- The sender composes the SMS with the keyword “ACC.”
- The proposed Java-based system collects the GSM location's parameters such as latitude, longitude, time stamp, and cell ID of the GSM location.
- The GSM's location parameters are passed to an SMS gateway, which forwards the SMS to the application server.
- The application server determines the origin/geolocation of the SMS and determines the rescue team closest to the origin.
- The server sends feedback and dispatches the rescue team.

The only visible drawback here for the user after the system was reviewed was the error generated from typos. Adding spaces in-between the keyword “ACC”; adding spaces after the keyword or mistakenly adding a symbol counts as a character and will generate exception errors which have not been handled within the system.

Figure 2 is a system stem architecture (pictorial illustration) of the proposed system showing the base station triangulation utilized by the mobile device sending the emergency notification with *X*/*Y* coordinates transmitted simultaneously.

### 3.1. Server Hardware and Software Requirements

The following hardware requirements must be met on the server:

- Core 2 duo computer system.
- Minimum of 2.80 GHz processing capacity.
- Minimum of 2 GB RAM system memory and 32-bit instruction set.
- 500 GB free hard disk space for various software installations.
- 64 MB DirectX9 and 3D capable graphics card.
- Resolution of 1024 × 768, 16-bit high color—DirectX9 (to run in DirectX mode).
- Uninterrupted power supply system with inverters.
- Internet connection.

Likewise, the following software must run on the server before it can serve the clients' needed services:

- Network-based operating system such as Windows 7, Linux, and Solaris.
- Web/application server software such as Apache, Internet Information System (IIS), web logic, web sphere, and Glassfish 3.1.1.
- Java virtual machine (JVM) interpreter to interpret the Java Servlet bytecodes to the web server software. Glassfish was utilized for this research work.
- An SMS gateway client software which may be web based or desktop version.

It is also of necessity that the geographical service area for the emergency geolocation notification system be defined and updated with images and street data using Panoramio web service on Google Map, that is the geographical area the system will cover.

### 3.2. Activity Diagram for the Proposed System

**Figure 3:**
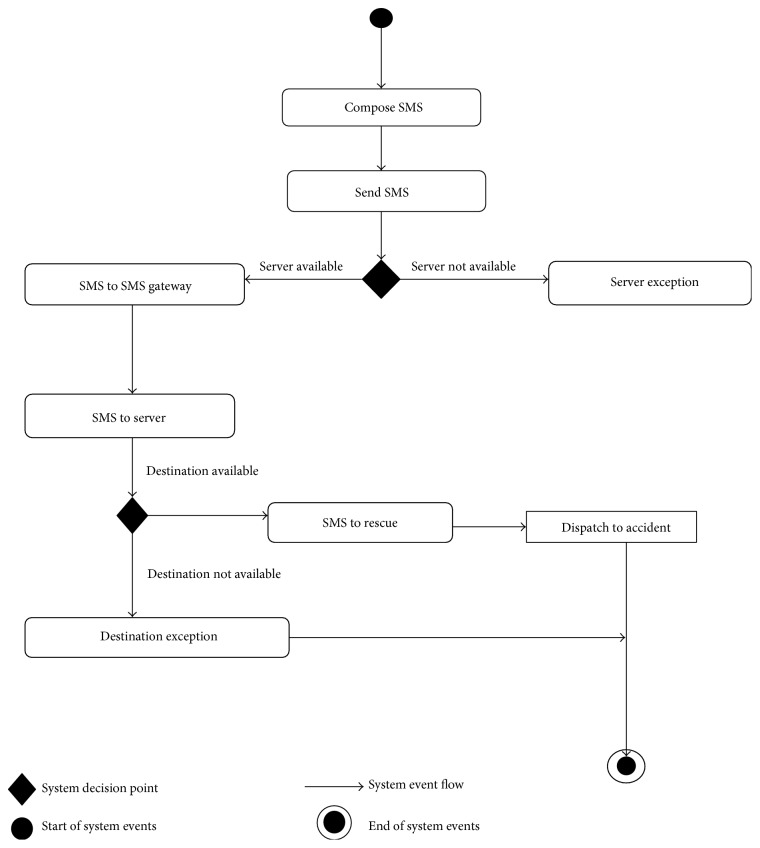
System activity diagram showing the movement and flow of activities from when the notification SMS is composed and sent.

Activity diagrams show graphically the workflows of stepwise activities and actions with support for choice, iteration, and concurrency.  Figure 3 is an activity diagram to show the movement and flow of activities of SMS. The SMS gateway receives the sent SMS and passes it to the server on availability. On availability too, the mapping information determined at the server component is passed to the dispatch team, which in turn responds by locating the accident scene using the mapping information transmitted.

## 4. System Evaluation, Interpretation, and Comparison

For the system being reported, thirteen thousand five hundred (13,500) SMSs with the key word “ACC” were sent and used as test runs during system deployment under normal conditions and situations. This figure was chosen to accommodate the emergency-scale traffic volume via SMS configured on the SMS gateway used within the system architecture. The SMSs were sent and received discreetly and concomitantly, all at different time of the day in a space of 5 days. System evaluation was carried out by considering two key parameters: the SMS delivery time and the system geolocation and dispatch time which form the needed elements to improve on if the amount of time it takes to get to the scene of an emergency also referred to as the time to scene (as explained in Section 2) is to be improved on. This time to scene represents one of the three different measures of the timeliness of ambulance response to the emergency scene as discussed in Section 2.1. For each SMS sent, the following parameters were recorded and used for the performance evaluation of the system:

- Time intervals between sending and receiving of SMS confirmation.
- Time in seconds between sending the SMS and receipt of confirmation (*x*_1_).
- Time in seconds taken by the system to determine the SMS origin/geolocation and to dispatch the closest ambulance to the scene (*x*_2_).
- Total time in seconds from when the SMS was sent and received to when dispatching occurred (*x*_1_ + *x*_2_).

The mean or average time in seconds it took the SMS to arrive at the control center was calculated using: ∑*fx*/∑*f* with four seconds being the benchmark for the system efficiency, effectiveness, and RAS rating and calculation. This benchmark is utilized as it has been concluded that a typical SMS delivery takes approximately four seconds in a GSM network [25]. Determining originating SMS geolocation and dispatching take approximately 8 seconds.

Evaluation on the system was carried out using the RAS benchmark metrics and model. These metrics can be applied to a range of software including application programs. The Institute of Electrical and Electronics Engineers (IEEE) sponsors an organization devoted to the reliability in engineering known as the IEEE Reliability Society (IEEE RS). A reliable system does not silently continue and deliver results that include uncorrected corrupted data. Instead, it detects and, if possible, corrects the corruption. This shows the likelihood that a system component will succeed within its identified mission time with no failures [26].

*Reliability* here is the measure of the system's ability to function correctly while avoiding data corruption. It is often characterized in terms of mean time between failures (MTBF).

(1) $$\begin{array}{c} Reliability=\text{exp}\left(\frac{-t}{MTBF}\right). \end{array}$$

The higher the MTBF value is, the higher the reliability of the system. Reliability is quantified as MTBF for repairable systems and mean time to failure (MTTF) for nonrepairable systems. The term reliability refers to the ability of a computer-related hardware or software component to consistently perform according to its specifications. In theory, a reliable product is totally free of technical errors. In practice, vendors commonly express product reliability as a percentage. This shows the likelihood that a system component will succeed within its identified mission time with no failures [26].

*Availability* on the other hand is the ratio of time a system or component is functional to the total time it is required or expected to function. This can be expressed as a direct proportion (e.g., 9/10 or 0.9) or as a percentage (e.g., 90%). It can also be expressed in terms of average downtime per week, month, or year or as total downtime for a given week, month, or year (see Table 1).

(2) $$\begin{array}{c} Availability=\frac{MTBF}{MTBF+MTTR}. \end{array}$$

*Serviceability (Maintainability)* is an expression of the ease with which a component, device, or system can be maintained and repaired. Some systems have the ability to correct problems automatically before serious trouble occurs. Mean time to repair (MTTR) is a basic measure of the maintainability of repairable items. It represents the average time required to repair a failed component or device.

### 4.1. SMS Delivery Time Evaluation

SMS delivery takes approximately 4 seconds in a GSM network [25].  Table 2 depicts a frequency, showing the delivery time analysis of 13,500 SMSs sent during the 5-day period of implementation.

(i) Overall mean time for SMS delivery in seconds computed from Table 2 is
  (3) $$\begin{array}{c} \frac{\sum {fx}_{1}}{\sum f}=\frac{54,107}{13,500}=4.01\;seconds. \end{array}$$

This mean value further validates the fact that the SMS service utilized within the application meets up with the established standard in literature. It only exceeds this standard by 0.01 seconds.

(ii) MTBF is computed by dividing the total time period the system was set up by the total number of times the delivery exceeded 4 seconds. The system here was set up for a period of 5 days (120 hours) which amounts to
  (4) $$\begin{array}{c} 120\;hours\times 1\;\left(the\;single\;component\;being\;tested\right)=120. \end{array}$$

Total frequency for SMS deliveries above 4 seconds (regarded as system failures) from Table 2 is 302 + 211 + 58 + 41 + 117 + 110 = 839:

(5) $$\begin{array}{c} MTBF=\frac{120\;hours}{839}=\frac{0.1430274136\;hours}{failure}. \end{array}$$

(iii) *Reliability* = *e*^−*t*/MTBF^ = 6.9916666667.

From literature, this amounts to a reliability of 69.9% ≈ 70.0% for the system.

This tells us that the probability that this particular component/module will survive to its calculated MTBF is 70.0%.

(iv) *Serviceability*: MTTR = 7 seconds (0.0019444444 hours) (total mean time for system reboot after fault detection).
(v) *Availability* = MTBF/(MTBF + MTTR).

MTBF = 0.1430274136 hours while MTTR = 0.0019444444 hours (7 seconds) (total mean time for system reload after fault detection).

(6) $$\begin{array}{c} \frac{MTBF}{MTBF+MTTR}=\frac{0.1430274136}{0.1430274136+0.0019444444}=0.9865874354\approx 0.99. \end{array}$$

This interprets to a 99% (2 nines) availability and deducing from Table 1. This system component has a downtime of 3.65 days/year. This means that the system will not be available for a total period of at most 3.65 days for an operational time of a year (365 days); this downtime comprises of the sum of scheduled and unscheduled downtime for the system. Also, the probability that this particular component/module will survive to its calculated MTBF is 70.0%. This is a little short of the systems required reliability performance of 100%, but this is acceptable since we are dealing with a repairable system that is comprised of other repairable and nonrepairable components [27]. To improve on this aggregate reliability value, the various components' reliability will need to be improved on individually, especially the least reliable and weak ones in the system. This is so because the least reliable or weak component has the biggest effect on system reliability.

### 4.2. System Geolocation and Dispatch Time Evaluation

Here, 8 seconds is taken as the benchmark for geolocation and dispatch time for the system.  Table 3 is a frequency table showing the dispatch time analysis of 13,500 simulations.

(i) Overall mean time for geolocation determination and dispatch in seconds from Table 3 is
  (7) $$\begin{array}{c} \frac{\sum {fx}_{2}}{\sum f}=\frac{108,069}{13,500}=8.01\;seconds. \end{array}$$

This mean value further validates the fact that the geolocation and dispatch components utilized within the application meet up with the set standard. It only exceeds by 0.01 seconds.

(ii) MTBF is computed by dividing the total time period the system was set up by the total number of times the delivery exceeded 8 seconds. The system here was set up for a period of 5 days (120 hours) which amounts to
  (8) $$\begin{array}{c} 120\;hours\times 1\;\left(the\;single\;component\;being\;tested\right)=120. \end{array}$$

Total frequency for geolocation and dispatch time above 8 seconds from Table 4 functionality 3 is 291 + 173 + 171 + 118 = 753:

(9) $$\begin{array}{c} MTBF=\frac{120\;hours}{753}=0.1593625498\;hours. \end{array}$$

(iii) *Reliability* = *e*^−*t*/MTBF^ = 6.275.

From literature, this amounts to a reliability of 62.7% per year.

This tells us that the probability that this particular component/module will survive to its calculated MTBF is only 62.7%.

(iv) *Serviceability*: MTTR = 9 seconds (0.002500000 hours) (total mean time for server reload after fault detection).
(v) *Availability* = MTBF/(MTBF + MTTR).

MTBF = 0.1593625498 hours while MTTR = 9 seconds (0.002500000 hours) (total mean time for server reload after fault detection):

(10) $$\begin{array}{c} \frac{MTBF}{MTBF+MTTR}=\frac{0.1593625498}{0.1593625498+0.0025000000}=0.98455479663\approx 0.99. \end{array}$$

This interprets to a 99% (2 nines) availability and deducing from Table 1; this system component has a downtime of 3.65 days/year. This means that the system will not be available for a period total of at most 3.65 days for an operational time of a year (365 days), this downtime comprises of the sum of scheduled and unscheduled downtime for the system. The calculated reliability which indicates the probability of survival of this particular component/module is 62.7%. As discussed in Section 4.1 , this low value is due to the fact that we are dealing with a repairable system that is comprised of other repairable and nonrepairable components [27].

### 4.3. Comparative Analysis

To check and ascertain that the designed system provides better emergency notification functionalities, a comparison with other similar existing systems was carried out as stated in Section 2. Our proposed system is strictly for users to notify ambulance points of emergencies with the sent SMS transmitting the geolocation of the sender automatically and invisibly. This makes it suitable and easy to use when making silent calls especially during risky emergencies as earlier mentioned.

#### 4.3.1. Case Selection

A selected number of systems that addresses emergency notification system with different emergency functionalities were reviewed in the literature survey. Our work is only limited to three in particular, MEHM-DESIGN [17] the emergency SMS [14], and POLINT-112-SMS [15], since only these were found to be similar in system design and setup. We are not including the other ones, for example, E-911, which is a GPS-based system.

The emergency SMS system design [14] requires the use of a GPS system to get locations, and this comes with all the drawbacks of the GPS system. Our designed system utilizes Google Map application programming interfaces (APIs) to translate base station triangulated coordinates where SMS is originating from, into a map. The advantage of this technology is that it has no obstructions; it can be used even in tunnels where GSM receptions are present.

The POLINT-112-SMS [15] does not clearly address emergency situations in its use. It is essentially designed to support information management and to assist a human in decision making in emergency situations. During emergencies, frightful users will find it difficult to compose long SMS that reports the crisis at hand. Our proposed system addresses this limitation.

In the SMS module of the MEHM-DESIGN [17], the nearest healthcare center is located based on provided inputs by user through an SMS request during an emergency. This module is required to communicate with Google geocoding service and local hospital database to gather the different healthcare center name, address, and contact information, retrieve them, and send by SMS to the requester or user. In risky emergencies, this is inadequate. Our designed system does not require the user to try to know the nearest healthcare center, but the nearest ambulance point will locate the user initiating the SMS using maps interpreted by the system from the user's cell triangulation position.

#### 4.3.2. Data Collection and Analysis Procedure

Table 4 presents a comparison between our designed system and the existing systems. It shows the benefits of our designed system according to a number of criteria which were selected according to the common features and functionalities required by an emergency notification system aimed at reducing ambulance response time. These are making silent calls during risky emergencies, automatic transmission of geolocation from an emergency scene by SMS to the nearest ambulance point automatically, ease of use and time-saving functionality during use, GPS technology required, SMS gateway to improve SMS reliability and delivery time, strictly designed for reporting emergencies in real time, and swift emergency geolocation transmission due to automatic geolocation transmission functionality.

#### 4.3.3. Result

Our designed system was implemented, evaluated, and interpreted as reported in Section 4. It meets up with the SMS delivery time of approximately 4 seconds in a GSM network [25] with an SMS mean delivery time of 4.01 seconds as reported in Section 4.1 and 8.01 seconds mean time for geolocation transmission and dispatch time for the system as reported in Section 4.2. The comparative analysis in Table 4 further proves that our proposed system is flexible, requires less time to use, provides reliable mapping data transmission, performs invisible and background geolocation transmission, and automatic system notification/response from the closest ambulance point.

The three reviewed systems were found to be capable of supporting information management between the patient and the doctor. Unlike the proposed system, they do not transmit geolocation but only support the sending of a location descriptively by patients reporting emergencies. This involves the composing of location information, emergency scene crisis report, or patient biodata in text form and sending by SMS. The proposed system only transmits geolocation from point of initiation to ambulance points and does not need to send any message back to the point of initiation but transmits the received geolocations to the nearest ambulance point.

The advantages of the proposed application that differentiates it from others are summarized as follows:

- The proposed application is highly effective when silence is nonnegotiable and voice call becomes dangerous when making an emergency call, especially during robbery, kidnapping, or gunshot attacks (silent calls).
- The proposed application is convenient for speech-impaired or hearing-impaired persons to report emergencies.
- The proposed application is also convenient in reporting emergency situations when the caller is not familiar with the neighborhood of the emergency scene since it transmits geolocations automatically by SMS.
- It can be used in reporting emergencies in real time.
- The proposed application can be used on all SMS capable mobile devices; it will still function even if the person to save the day does not have a smart phone or a mobile device with LBS capabilities.

### 4.4. Pros, Cons, and Limitations

The advantage of the proposed system is that it uses the GSM base station triangulation to deliver location coordinates so it can be used even in tunnels where GSM receptions are present. The system has also been found useful in getting a location from persons experiencing panic or fear in situations where the person making help calls on the phone is a close relative of a dying patient. Also, it is so useful when silence is nonnegotiable and voice call becomes dangerous when making an emergency call, especially during robbery, kidnapping, or gunshot attacks. In addition, it is recommended for dumb (speech-impaired) or deaf (hearing-impaired) persons in reporting emergencies.

A disadvantage of this system is its inability to interpret and deliver geolocation parameters when the server is down; this means that power supply to the server must be at all times. This makes the system a little expensive to maintain in this part of the world.

The RAS (reliability, availability, and serviceability) metrics were picked for the system evaluation and analysis because of the presence of some additional nonfunctional requirements since the system requires minimal human intervention. This is recognized as a limitation for this work since other software performance evaluation approaches exist. These other approaches that will be employed in the future to further improve this work. As a limitation also, this work did not cover the cost-benefit analysis for the system or other factors such as the business and security aspects. Some elements such as sensitivity, benefits, cost, specificity, and user satisfaction were not considered for this work, also as it was observe that they had inconsequential effects on the performance of the proposed system.

## 5. Conclusion and Future Works

We have proposed an SMS-based mobile application suitable for emergency situations. After testing and evaluating its performance, the designed system was found to be efficient and effective as its reliability stood within 62.7% to 70.0% while its availability stood at 99% with a downtime of 3.65 days/year. This shows that the effective system was effective in use in this geographic region better than the existing systems.

For future works, near field technology and the GPS technology can be combined with the GSM localization to improve transmissions and communication of geolocations with no geographical restrictions. Also, this application can be adapted for use in fire service departments for fire emergencies and for accident notification on highways. We also aim to evaluate our system against some selected evaluation approaches, one of which is the prediction-enabled component technology (PECT). The RAS metrics used in this paper provide a single reliability value for the entire system component, but PECT will be engaged to provide reliability information about each role that various system components are intended to support. PECT is used to predict the reliability of a component assembly from the reliabilities of the individual components. This can then lead to computing the effective reliability during usage of the component. The main intent of PECT is to provide useful information about the reliability of various components of a system for the prediction of assembly reliability.

## Figures and Tables

**Table 1:** Table used for deductions that compares the availability and the corresponding downtime [28].

Availability	Downtime
90% (1 nine)	36.5 days/year
99% (2 nines)	3.65 days/year
99.9% (3 nines)	8.76 hours/year
99.99% (4 nines)	52 minutes/year
99.999% (5 nines)	5 minutes/year
99.9999% (6 nines)	31 seconds/year

**Table 2:** Frequency table showing the delivery time analysis of the 13,500 SMSs sent during implementation.

Time in seconds between sending SMS and receipt of confirmation (*x*_1_)	Frequency for each time recorded (*f*)	*f* × *x*_1_
3	2526	7578
4	10,135	40,540
5	302	1510
6	211	1266
8	58	464
9	41	369
10	117	1170
11	110	1210
	∑*f* = 13,500	∑*fx*_1_ = 54,107

**Table 3:** Frequency table showing the dispatch time analysis for the 13,500 simulations.

Time in seconds taken by system to determine SMS origin geolocation and dispatch closest ambulance to scene (*x*_2_)	Frequency for each time recorded (*f*)	*f* × *x*_2_
6	787	4722
7	622	4354
8	11,338	90,704
9	291	2619
10	173	1730
12	171	2052
16	118	1888
	∑*f* = 13,500	∑*fx*_2_ = 108,069

**Table 4:** A comparison of the proposed SMS notification application with others.

Functionalities	Emergency SMS [14]	POLINT-112-SMS [15]	MEHM-DESIGN [17]	The proposed application
(1) Making silent calls during risky emergencies	Yes	Yes	Not applicable	Yes
(2) Automatic transmission of geolocation from an emergency scene by SMS to the nearest ambulance point automatically	No	No	No	Yes
(3) Ease of use and time-saving functionality during use	Yes	No	Partially	Yes
(4) GPS technology required	Yes	No	No	No
(5) SMS gateway to improve SMS reliability and delivery time	No	Yes	Uses telephony server daemon	Yes
(6) Strictly designed for reporting emergencies in real time	Yes	Partially	No	Yes
(7) Swift emergency geolocation transmission due to automatic geolocation transmission functionality	No	No	No	Yes
(8) Has information management support capability	Yes	Yes	Yes	Partially
(9) Receives patients' bio, medical, and scene crisis report data by SMS	Yes	Yes	No	No
(10) Sends healthcare-related information apart from geolocation to the patients/users	Yes	No	Yes	No
